# Topographic Relationship between the Supratrochlear Nerve and Corrugator Supercilii Muscle—Can This Anatomical Knowledge Improve the Response to Botulinum Toxin Injections in Chronic Migraine?

**DOI:** 10.3390/toxins7072629

**Published:** 2015-07-17

**Authors:** Hyung-Jin Lee, Kwang-Seok Choi, Sung-Yoon Won, Prawit Apinuntrum, Kyung-Seok Hu, Seong-Taek Kim, Tanvaa Tansatit, Hee-Jin Kim

**Affiliations:** 1 Division in Anatomy and Developmental Biology, Department of Oral Biology, Human Identification Research Center, BK21 PLUS Project, Yonsei University College of Dentistry, 50 Yonsei-ro, Seodaemun-gu, Seoul 120-749, Korea; E-Mails: leehj221@yuhs.ac (H.-J.L.); gattaca04@hanmail.net (K.-S.C.); hks318@yuhs.ac (K.-S.H.); 2 Department of Occupational Therapy, Semyung University, Semyungro 65, Jecheonsi, Chungcheongbuk-do 390-711, Korea; E-Mail: apolius@naver.com; 3 Department of Oral Medicine, Temporomandibular Joint and Orofacial Pain Clinic, Yonsei University College of Dentistry, 50 Yonsei-ro, Seodaemun-gu, Seoul 120-749, Korea; E-Mail: k8756050@yuhs.ac; 4 The Chula Soft Cadaver Surgical Training Center and Department of Anatomy, Faculty of Medicine, Chulalongkorn University, 254 Phyathai Road, Patumwan 10330, Bangkok, Thailand; E-Mail: cyj7797@naver.com

**Keywords:** supratrochlear nerve, corrugator supercilii muscle, chronic migraine, trigger point injection, periorbital region

## Abstract

Chronic migraine has been related to the entrapment of the supratrochlear nerve within the corrugator supercilii muscle. Recently, research has shown that people who have undergone botulinum neurotoxin A injection in frontal regions reported disappearance or alleviation of their migraines. There have been numerous anatomical studies conducted on Caucasians revealing possible anatomical problems leading to migraine; on the other hand, relatively few anatomical studies have been conducted on Asians. Thus, the aim of the present study was to determine the topographic relationship between the supratrochlear nerve and corrugator supercilii muscle in the forehead that may be the cause of migraine. Fifty-eight hemifaces from Korean and Thai cadavers were used for this study. The supratrochlear nerve entered the corrugator supercilii muscle in every case. Type I, in which the supratrochlear nerve emerged separately from the supraorbital nerve at the medial one-third portion of the orbit, was observed in 69% (40/58) of cases. Type II, in which the supratrochlear nerve emerged from the orbit at the same location as the supraorbital nerve, was observed in 31% (18/58) of cases.

## 1. Introduction

The supratrochlear nerve (STN) and supraorbital nerve (SON) constitute the terminal branch of the frontal nerve, which is a major branch of the ophthalmic nerve. After passing over the roof of the orbit anteromedially, the STN and SON travel through the corrugator supercilii muscle (CSM) and proceed to the frontal belly of the occipitofrontalis muscle to receive the sensation of the mucosa of the glabella, upper eyelid, and skin of the lower forehead close to the midsagittal line [1,2].

The STN and SON are anesthetized prior to performing various surgical procedures [3,4]. For example, the nerve-block procedure and botulinum toxin type A (BTX-A) injection can be beneficial for treating primary headache disorder [5,6,7,8,9,10,11,12,13,14,15]. BTX-A is routinely administered as a treatment for chronic migraine in the frontal region [9,13,15]. The trigger point is generally targeted as the injection site for the BTX-A treatment to relieve the associated pain [16,17]. In the frontal region, chronic migraine may occur as a result of entrapment of the STN by the CSM; the STN could also be compressed by hyperactivity of the CSM [5,9]. Since the STN is thought to be a trigger point for headache in the frontal region [18,19], BTX-A injection is broadly performed in the supraorbital area. However, there has been little research to determine the most effective BTX-A injection point based on detailed knowledge of the anatomical structure of the frontal area.

In the USA, this nerve-block procedure and trigger-point injections carried out with lidocaine or bupivacaine have provided sustained and often rapid pain relief to patients experiencing various types of headache [20,21]. It has been recommended that anesthetics should be injected into the medial portion of the eyebrow to produce a successful nerve block of the STN [14]. However, while it is quite easy to find surface landmarks to enable SON block, it is quite difficult to find appropriate surface landmarks to enable anesthetization of the STN, despite the numerous studies describing the morphology of that nerve. This has resulted in numerous unnecessary injections to induce STN anesthesia in the forehead skin, and when it is achieved; this anesthesia is less effective compared with SON block [12,22,23,24]. Only a few of the studies that have investigated the STN morphology have described its patterns of emergence from the orbit and its course through the CSM. Patterns of emergence of the STN from the orbit are clinically important. Thus, elucidating the potential inter-individual differences in the anatomy and topography of the STN in the orbit, would aid the effective anesthesia of that nerve.

The aim of the present study was to determine the topographic relationship between the STN and CSM in the forehead region and to classify the patterns of emergence of the STN from the orbit and its course within the CSM.

## 2. Results

**Figure 1:**
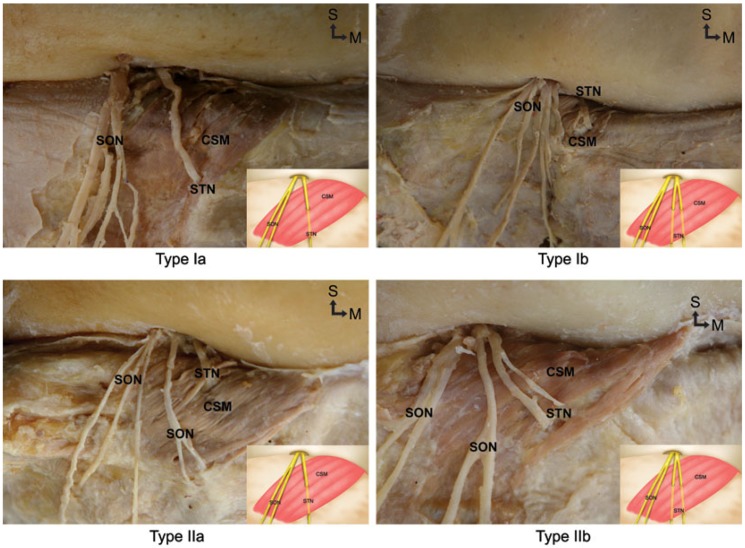
Photographs and schematic illustrations indicating the distribution patterns of the supratrochlear nerve (STN) to the corrugator supercilii muscle (CSM). In types Ia and Ib, the STN emerged independently from the supraorbital notch region, and then passed through the CSM either as a single nerve branch (type Ia) or after bifurcating into two branches prior to piercing the CSM (type Ib). In types IIa and IIb, the STN emerged from the supraorbital notch region with the supraorbital nerve (SON), and then passed through the CSM either as a single nerve branch (type IIa) or after bifurcating into two branches prior to piercing the CSM (type IIb) (S: superior, M: medial).

The emergence patterns of the STN were divided into two main types: I and II. The STNs typically emerged from the medial one-third portion of the orbit, running through the thin fatty tissue behind the orbicularis oculi muscle, and then entered CSM in every case. Type I, in which the STN emerged separately at the medial one-third portion of the orbit, was observed in 40 cases (69%). These cases could be further divided into type Ia, in which the STN entered the CSM as a single nerve branch, and type Ib, in which the STN bifurcated prior to entering the CSM. Types Ia and Ib were observed in 22 (38%) and 18 (31%) cases, respectively (Figure 1).

Type II, in which the STN emerged from the orbit at the same location as the SON, was observed in 18 cases (31%). As with type I, the type II category could be further divided into two subtypes: type IIa (13 cases, 22.4%), where the STN entered the CSM as a single nerve branch, and type IIb (5 cases, 8.6%), where the STN bifurcated into two branches prior to entering the CSM (Figure 1). In one case an accessory foramen was found medial to the supraorbital notch; a single nerve emerged from this exit and then entered the CSM. An extra exit for the STN was located 5 mm lateral to the midsagittal line.

**Figure 2:**
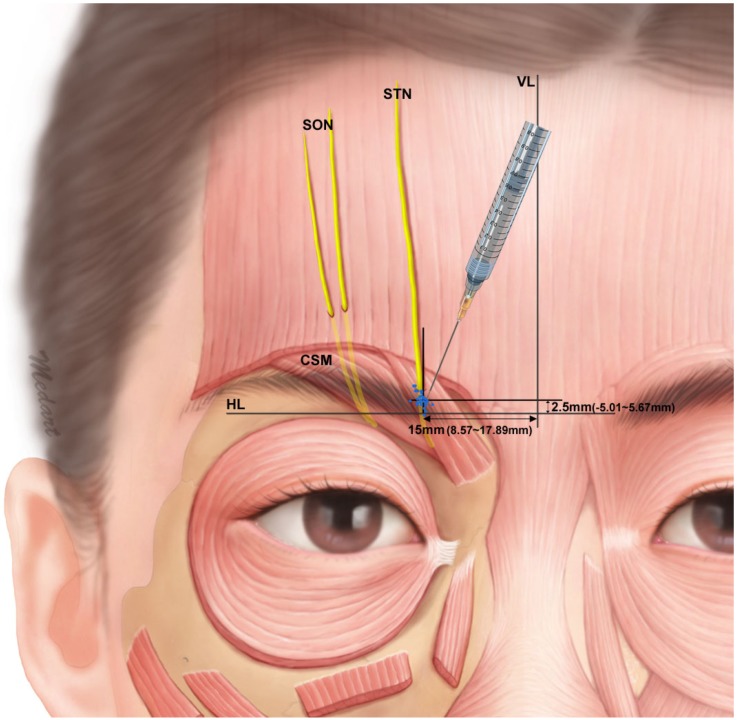
Location of the STN in the supraorbital notch region and within the CSM. Chronic migraine may develop in the location indicated by blue dots. (VL: vertical reference line, HL: horizontal reference line).

The point at which the STN entered the CSM was measured in every case, and the distance between the SON and STN in type I was measured. The point at which the STN entered the CSM was 16.4 ± 4.0 mm (mean ± SD) lateral to the midsagittal line and 2.3 ± 3.9 mm superior to the supraorbital margin. When the STN emerged separately from the orbit, it was located 7.5 ± 2.3 mm medial to the SON at the level of the supraorbital margin (Figure 2).

## 3. Discussion

Some anatomical studies have documented the exit point of the STN from the orbit, the relationship between the SON and STN, and the point at which these nerves enter the CSM, with a focus on clinical applications [12,21,23,24]. The present study revealed the branching patterns of the STN in the supraorbital area.

The STN and SON have been target nerves for nerve-block procedures to treat various types of headache and for local anesthesia before performing endoscopic and transpalpebral approaches in the forehead region [3,4]. The present study identified a previously unreported type of STN (type II), which was observed in 31% of the specimens, and which could be further classified into two subtypes (types IIa and IIb).

In contrast, Andersen *et al.* [22] reported that the STN exited the orbit as a single branch in 25% of cases, but was composed of two or three branches in the other cases. In addition, Miller *et al.* [24] found that the STN comprised one or more branches in 30% of cases, where that nerve exited from the orbit medial to the SON. The findings of the present and previous studies indicate that in addition to appearing as a single nerve branch, the STN can also exist as two or more branches innervating the midforehead skin.

In addition, the distance between the STN and SON was measured at the level of the supraorbital margin. The STN was situated 7.5 ± 2.3 mm medial to the supraorbital notch or the foramen along the supraorbital margin. In addition, the point of entrance of the STN into the CSM was 16.4 ± 4.0 lateral to the midsagittal line and 2.3 ± 3.9 mm superior to the supraorbital margin. The branch of the STN nearest to the midsagittal line was located between 8.5 and 26.7 mm from it in the present study; Andersen *et al.* [22] observed that this distance ranged between 8 and 30 mm. 

The relationship between the STN and CSM is important for a tension-type headache, because frontally localized headaches can be associated with nerve entrapment and compression of the peripheral nerve [9,18,25]. However, only a small percentage of migraine with nerve entrapment may be due to nerve entrapment. Yet, the STN and SON are thought to be the trigger spots in the frontal region [5,9,11,12,25]. Since the STN runs through the CSM in the midforehead area, it can be entrapped by contraction of the CSM [5,9]. BTX-A injection is used to treat chronic migraine [10,26,27,28,29,30]; According to Blumenfeld *et al.*, they performed two injections using 5 units on each side of the CSM for the BTX-A injection into the CSM. The injection point is set 15 mm above the medial superior edge of the orbital ridge. However, there is a discrepancy in the location of the CSM and STN between Caucasians and Asians and it is inappropriate to apply the same techniques to Asians. Thus, it is necessary to compare the location (or position) of these structures to treat chronic migraine effectively. 

Janis *et al.* stated the STN entered the CSM approximately 15 mm superior to the supraorbital margin and 19 mm lateral to the midsagittal line [12]. In the present study the point of entrance of the STN into the CSM was 2 mm superior to the supraorbital margin and 16 mm lateral to the midsagittal line. Based on these results, Asians and Caucasians have different anatomical morphology of the STN piercing point to the CSM. In addition, Janis *et al.* found that in 84% of Caucasians, the STN entered the CSM; however, in the present study, the STN penetrated the CSM in all cases. The STN entered the CSM as a single nerve branch in type Ia and IIa; the STN bifurcated into two branches prior to entering the CSM in type Ib and IIb. These results show that it is unclear whether Caucasians would be less morphologically inclined to suffer from intramuscular compression than Asians. Furthermore, Asian patients with type Ib and IIb morphological patterns are more likely to suffer from chronic migraine than patients with Ia and IIa morphological patterns due to entrapment of nerve branches.

In general, the BTX-A injection has been recommended as 5 units per corrugator supercilii muscle according to PREEMPT trial [8]. In the present study, the piercing point of the STN into the CSM was measured. Side effects are dose related in botulinum toxin therapy. We propose that injecting a lower dose (for example 2.5 units) in our defined anatomical site may produce the same therapeutic effect with a safer side effect profile. However, clinical trials using such lower doses are necessary in order to confirm or refute the utility of injection at this anatomical point.

In summary, in addition to the descriptions of the STN provided in anatomy textbooks, there are cases in which the nerve branches of the STN exit from the orbit along with the SON. Moreover, the STN could comprise either one or two branches entering the CSM. Given the different nerve patterns of the STN, it can be concluded that this nerve supplies the sensation to the medial part of the forehead as one or two branches, and that it may not only have a different exit point, but also the same exit point from the orbit. As show in the results, the STN’s path through the CSM was classified into four types (Ia, Ib, IIa, IIb). Further research should focus on the clinical significance of the anatomical variations among the four types.

## 4. Materials and Methods

**Figure 3:**
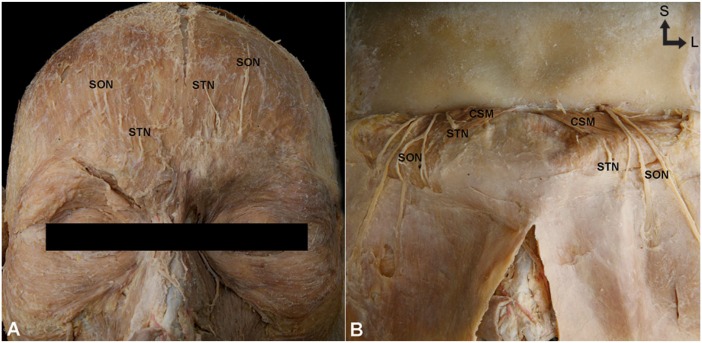
Frontal view of the intact specimen (**A**) and after reflecting the scalp over toward the orbit (**B**) (S: Superior, L: Lateral). SON: supraorbital nerve; STN: supratrochlear nerve; CSM: corrugator supercilii muscle.

Fifty-eight hemifaces from Korean (22 hemifaces, 9 left and 13 right; mean age, 71.8 years) and Thai (36 hemifaces, 17 left and 19 right; mean age 73.4 years) cadavers were used for this study. An incision was made at the coronal suture region. After reflecting the forehead scalp toward the orbit, a detailed periosteum dissection was conducted, taking extreme care not to damage the underlying frontal belly of the occipitofrontalis muscle, STN, SON, and CSM (Figure 3). The anatomical relationship between the STN and CSM was observed on the dissected specimens.

To indicate the point where the STN enters the CSM, the midsagittal line and the line connecting both supraorbital margins were used as the vertical and horizontal reference lines, respectively. We used these two reference lines when measuring the distance between the entry points of the STN and CSM using digital calipers (catalog No. 500-196-20, Mituyoto, Kanagawa, Japan).

**Figure 4:**
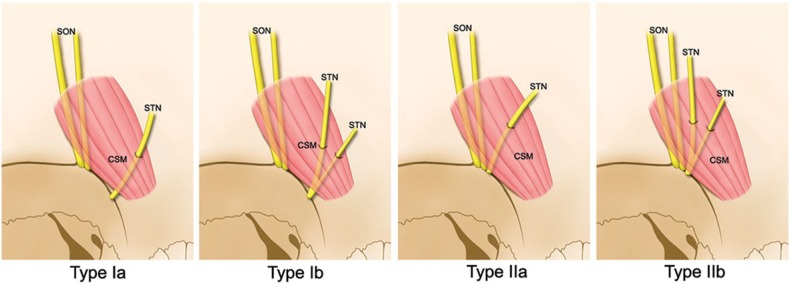
Schematic illustrations showing the four patterns of the STN within the CSM.

The course of the STN within the CSM was classified into the following four categories based on the type of emergence of the STN from the orbit (Figure 4):

Type Ia: The STN emerges independently from the supraorbital notch region, and passes through the CSM as a single nerve branch.
Type Ib: The STN emerges independently from the supraorbital notch region, and bifurcates into two branches prior to entering the CSM.
Type IIa: The STN emerges from the supraorbital notch region with the SON and passes through the CSM as a single nerve branch.
Type IIb: The STN emerges from the supraorbital notch region with the SON and then bifurcates into two branches prior to entering the CSM.

## 5. Conclusions

The findings of the present study indicate that in addition to appearing as a single nerve branch, the STN can also exist as two or more branches innervating the midforehead skin. The point at which the STN enters the CSM can be regarded as a trigger point in the frontal region. 

## References

[1] Moore K.L., Dalley A.F. (2006). Clinically Oriented Anatomy.

[2] Standring S., Gray H. (2008). Gray’s Anatomy the Anatomical Basis of Clinical Practice.

[3] Afifi A.M., Alghoul M., Zor F., Kusuma S., Zins J.E. (2012). Comparison of the transpalpebral and endoscopic approaches in resection of the corrugator supercilii muscle. Aesthet. Surg. J..

[4] Bidros R.S., Salazar-Reyes H., Friedman J.D. (2010). Subcutaneous temporal browlift under local anesthesia: A useful technique for periorbital rejuvenation. Aesthet Surg. J..

[5] Behmand R.A., Tucker T., Guyuron B. (2003). Single-Site botulinum toxin type a injection for elimination of migraine trigger points. Headache.

[6] Ducic I., Felder J.M., Fantus S.A. (2014). A systematic review of peripheral nerve interventional treatments for chronic headaches. Ann. Plast. Surg..

[7] Robbins M.S., Kuruvilla D., Blumenfeld A., Charleston L., Sorrell M., Robertson C.E., Grosberg B.M., Bender S.D., Napchan U., Ashkenazi A. (2014). Trigger point injections for headache disorders: Expert consensus methodology and narrative review. Headache.

[8] Liberini P., Pari E., Gazzina S., Caratozzolo S., Rao R., Padovani A. (2014). Technique of injection of onabotulinumtoxin A for chronic migraine: The PREEMPT injection paradigm. Neurol. Sci..

[9] De Ru J.A., Buwalda J. (2009). Botulinum toxin A injection into corrugator muscle for frontally localised chronic daily headache or chronic tension-type headache. J. Laryngol. Otol..

[10] Dodick D., Blumenfeld A., Silberstein S.D. (2004). Botulinum neurotoxin for the treatment of migraine and other primary headache disorders. Clin. Dermatol..

[11] Guyuron B., Varghai A., Michelow B.J., Thomas T., Davis J. (2000). Corrugator supercilii muscle resection and migraine headaches. Plast Reconstr. Surg..

[12] Janis J.E., Hatef D.A., Hagan R., Schaub T., Liu J.H., Thakar H., Bolden K.M., Heller J.B., Kurkjian T.J. (2013). Anatomy of the supratrochlear nerve: Implications for the surgical treatment of migraine headaches. Plast Reconstr. Surg..

[13] Kim C.C., Bogart M.M., Wee S.A., Burstein R., Arndt K.A., Dover J.S. (2010). Predicting migraine responsiveness to botulinum toxin type A injections. Arch. Dermatol..

[14] Levin M. (2010). Nerve blocks in the treatment of headache. Neurotherapeutics.

[15] Grazzi L. (2013). Onabotulinum toxin A for treatment of chronic migraine with medication overuse. Neurol. Sci..

[16] Gerwin R. (2011). Treatment of chronic migraine headache with onabotulinumtoxinA. Curr. Pain Headache Rep..

[17] Benecke R., Heinze A., Reichel G., Hefter H., Gobel H., Dysport myofascial pain study group (2011). Botulinum type A toxin complex for the relief of upper back myofascial pain syndrome: How do fixed-location injections compare with trigger point-focused injections?. Pain Med..

[18] Guyuron B., Kriegler J.S., Davis J., Amini S.B. (2005). Comprehensive surgical treatment of migraine headaches. Plast Reconstr. Surg..

[19] Fallucco M., Janis J.E., Hagan R.R. (2012). The anatomical morphology of the supraorbital notch: Clinical relevance to the surgical treatment of migraine headaches. Plast Reconstr. Surg..

[20] Afridi S.K., Shields K.G., Bhola R., Goadsby P.J. (2006). Greater occipital nerve injection in primary headache syndromes--prolonged effects from a single injection. Pain.

[21] Blumenfeld A., Ashkenazi A., Grosberg B., Napchan U., Narouze S., Nett B., DePalma T., Rosenthal B., Tepper S., Lipton R.B. (2010). Patterns of use of peripheral nerve blocks and trigger point injections among headache practitioners in the USA: Results of the American Headache Society Interventional Procedure Survey (AHS-IPS). Headache.

[22] Andersen N.B., Bovim G., Sjaastad O. (2001). The frontotemporal peripheral nerves. Topographic variations of the supraorbital, supratrochlear and auriculotemporal nerves and their possible clinical significance. Surg. Radiol. Anat..

[23] Konofaos P., Soto-Miranda M.A., Ver Halen J., Fleming J.C. (2013). Supratrochlear and supraorbital nerves: An anatomical study and applications in the head and neck area. Ophthal Plast. Reconstr. Surg..

[24] Miller T.A., Rudkin G., Honig M., Elahi M., Adams J. (2000). Lateral subcutaneous brow lift and interbrow muscle resection: Clinical experience and anatomic studies. Plast Reconstr. Surg..

[25] Janis J.E., Ghavami A., Lemmon J.A., Leedy J.E., Guyuron B. (2008). The anatomy of the corrugator supercilii muscle: Part II. Supraorbital nerve branching patterns. Plast Reconstr. Surg..

[26] Aurora S.K., Dodick D.W., Diener H.C., DeGryse R.E., Turkel C.C., Lipton R.B., Silberstein S.D. (2014). OnabotulinumtoxinA for chronic migraine: Efficacy, safety, and tolerability in patients who received all five treatment cycles in the PREEMPT clinical program. Acta Neurol. Scand..

[27] Aurora S.K., Dodick D.W., Turkel C.C., DeGryse R.E., Silberstein S.D., Lipton R.B., Diener H.C., Brin M.F., PREEMPT 1 Chronic Migraine Study Group (2010). OnabotulinumtoxinA for treatment of chronic migraine: Results from the double-blind, randomized, placebo-controlled phase of the PREEMPT 1 trial. Cephalalgia.

[28] Aurora S.K., Winner P., Freeman M.C., Spierings E.L., Heiring J.O., DeGryse R.E., VanDenburgh A.M., Nolan M.E., Turkel C.C. (2011). OnabotulinumtoxinA for treatment of chronic migraine: Pooled analyses of the 56-week PREEMPT clinical program. Headache.

[29] Dodick D.W., Turkel C.C., DeGryse R.E., Aurora S.K., Silberstein S.D., Lipton R.B., Diener H.C., Brin M.F., PREEMPT Chronic Migraine Study Group (2010). OnabotulinumtoxinA for treatment of chronic migraine: Pooled results from the double-blind, randomized, placebo-controlled phases of the PREEMPT clinical program. Headache.

[30] Ashkenazi A., Silberstein S. (2008). Botulinum toxin type A for the treatment of headache: Why we say yes. Arch. Neurol..

